# Selection of DNA Aptamers Against Parathyroid Hormone for Electrochemical Impedimetric Biosensor System Development

**DOI:** 10.1002/bab.2745

**Published:** 2025-03-19

**Authors:** Reza Didarian, Saharnaz Bargh, Almina Gülerman, Veli Cengiz Özalp, Özcan Erel, Nimet Yildirim‐Tirgil

**Affiliations:** ^1^ Department of Biomedical Engineering Ankara Yıldırım Beyazıt University Ankara Türkiye; ^2^ Department of Radiopharmacy, Faculty of Pharmacy Hacettepe University Ankara Türkiye; ^3^ Department of Metallurgy and Materials Engineering Ankara Yıldırım Beyazıt University Ankara Türkiye; ^4^ Department of Medical Biology, School of Medicine Atılım University Ankara Türkiye; ^5^ Department of Biovchemistry, Faculty of Medicine Ankara Yıldırım Beyazıt University Ankara Türkiye

**Keywords:** aptamer, electrochemical detection, electrochemical impedance spectroscopy (EIS), impedimetric biosensor, parathyroid hormone (PTH), SELEX

## Abstract

This work presents the pioneering development of an aptamer‐based electrochemical biosensor for real‐time monitoring of parathyroid hormone (PTH) levels, with a focus on intraoperative assessment during parathyroid surgery. It introduces, for the first time, the selection and characterization of aptamers targeting distinct segments of the PTH peptide. The study demonstrates the feasibility and efficacy of the biosensing platform through a precisely designed experimental framework, including SELEX‐based aptamer selection, aptamer–peptide interaction analysis, and biosensor fabrication. The SELEX process yields aptamers with notable binding affinities to different fragments of PTH, with the PTH (53–84) aptamer showing particularly sensitive binding to the hormone's C terminus, allowing for precise PTH analysis. Electrochemical characterization reveals significant changes in electrochemical impedance spectroscopy (EIS) signals upon exposure to varying PTH concentrations, highlighting the sensor's sensitivity and selectivity. The increase in charge transfer resistance (Rct) values with rising PTH concentrations underscores the biosensor's capability to detect PTH‐induced structural changes, validating its potential for accurate measurement. The biosensor shows remarkable selectivity in the presence of common interferents in serum samples, ensuring precise PTH detection. Stability assessments over a 45‐day storage period demonstrate the biosensor's robustness and long‐term reliability, affirming its practical suitability. In summary, the developed aptamer‐based biosensor represents a promising tool for sensitive and selective PTH detection, with potential applications in biomedical research and clinical diagnostics, particularly for intraoperative PTH analysis during parathyroidectomy. Continued research and optimization efforts hold promise for enhancing its performance and expanding its utility in diverse healthcare settings.

## 1 Introduction

Parathormone, often abbreviated as PTH, is a vital hormone produced by the parathyroid glands, four small pea‐sized glands located in the neck adjacent to the thyroid gland [1]. PTH plays a central role in regulating the balance of calcium and phosphorus in the body, making it indispensable for various physiological processes [2]. The primary function of PTH is to increase the concentration of calcium in the bloodstream by stimulating the release of calcium from bone tissue, enhancing the reabsorption of calcium in the kidneys, and promoting the production of active vitamin D in the kidneys, which in turn aids in the absorption of calcium from the intestines [3]. This delicate hormonal control of calcium levels is essential for muscle and nerve function, bone health, and various other cellular processes. An imbalance in PTH levels can lead to disorders such as hyperparathyroidism (excess PTH production) or hypoparathyroidism (insufficient PTH production), both of which have significant clinical implications [4].

It is known that PTH is associated with one of the most common diseases: primary hyperparathyroidism (pHPT). pHPT is an endocrine disorder characterized by excessive secretion of PTH, which can lead to disruptions in the regulation of calcium metabolism. Hyperparathyroidism poses significant clinical challenges, potentially resulting in various health issues for patients, such as weakness, abdominal pain, mental disorders, gastric ulcers, pancreatitis, and osteoporosis [1, 5]. One of the most effective treatments for this disease involves the surgical removal of pathological parathyroid glands, a procedure known as parathyroidectomy. An essential tool in managing this condition, especially in the context of minimally invasive parathyroidectomy, is the real‐time monitoring of intraoperative parathyroid hormone (ioPTH) levels. This monitoring allows surgeons to assess the success of the surgery by determining the completeness of gland excision. Given the biological half‐life of intact PTH, which is approximately 2–5 min, a specific reduction in PTH levels after the removal of parathyroid glands indicates the success of the surgery [6, 7, 8]. Several studies have shown that a single intraoperative rapid PTH measurement during surgery can change the operative treatment in 17% of patients with a single adenoma, increasing the surgical success rate to 97% [9]. However, in the case of double adenomas, the success of a single test may decrease, and that is why some centers take two separate samples at intervals of 5–10 min. If the expected drop is not achieved, the surgery continues to investigate the presence of a second adenoma [10]. On the other hand, obtaining ioPTH results can sometimes be time‐consuming. Literature review reveals that the average time for obtaining ioPTH results compared to standard laboratory tests is approximately 41.5 min [11]. However, this duration may vary depending on the test devices used in laboratory settings, and it may also necessitate the transportation of samples from the operating room to the laboratory. Therefore, the rapid and reliable measurement of PTH levels is of utmost importance, not only for shortening the duration of the surgery but also for assisting patients in achieving a faster recovery.

Numerous platforms have been developed for ioPTH measurement, including routine biochemical tests. These platforms share a similar three‐step analytical principle: capturing the PTH molecule within a specific sample by recognizing a particular amino acid sequence within the PTH peptide using specific antibodies. Detecting the captured PTH molecule using signal‐labeled specific secondary antibodies. Reading the signal intensity in conjunction with determining the quantity of captured PTH within the sample. The antibodies used are typically polyclonal antibodies with binding properties to both the entire PTH molecule and the PTH‐C fragment. Signal generation is often achieved using chemiluminescent labels. However, there are some differences among these platforms concerning the use of solid or liquid phases and incubation conditions, leading to variations in the required sample volume, time for obtaining results, and measurement range [12, 13, 14]. These general laboratory analyses, while offering sufficient detection capabilities for PTH level analysis, come with disadvantages such as lengthy testing times (ranging from one to several hours), the need for well‐equipped and expensive laboratory equipment, and the necessity to transport samples to the laboratory. The use of non‐portable devices further adds to the challenges in laboratory settings. Apart from these systems, there are also biosensor systems for PTH analysis where antibodies are employed as bio‐recognition agents. Particularly, biosensor systems based on electrochemical detection provide the capability to measure PTH with high sensitivity and selectivity [15, 16, 17]. These systems, offering rapid and partially cost‐effective analysis, also have various limitations due to the nature of antibodies. For instance, the use of antibodies requires high sensitivity, making them sensitive to factors like temperature, pH, and organic compounds. Additionally, their production involves the use of bacteria, animals, and so forth, leading to extra costs and lengthy processes [18, 19]. For these reasons, developing an analysis system that can rapidly and accurately detect target molecules does not rely on antibodies or peptide‐based recognition molecules, and can be used within the operating room for point‐of‐care testing, which is of great importance for ioPTH detection. Such new systems, if they can overcome the limitations of general laboratory analyses, enable faster results, thereby reducing surgical duration and providing time and cost savings.

Aptamers have garnered considerable attention in the field of bioidentification, offering distinct advantages over traditional antibodies. These single‐stranded molecules, often composed of DNA, RNA, or modified nucleic acids, have the unique ability to fold into intricate three‐dimensional structures, allowing them to specifically bind to a wide array of target molecules, including small chemicals, proteins, and even cells, facilitating recognition [20, 21]. This binding capability is achieved through a highly efficient in vitro selection process known as the Systematic Evolution of Ligands by Exponential Enrichment (SELEX) [20, 22]. One of the primary advantages of aptamers over antibodies lies in their in vitro selection process. Unlike antibodies, which are typically raised in animals, aptamers are entirely developed within a controlled laboratory environment, eliminating the need for animal immunization and providing a more ethical and sustainable approach to bioidentification [21, 23].

Aptamers exhibit a remarkable degree of specificity and affinity for their target molecules, often rivaling or surpassing the binding capabilities of antibodies [24]. Their smaller size and flexibility enable them to target epitopes that may be inaccessible to antibodies. Additionally, aptamers can be easily modified with various functional groups, such as fluorescent and redox molecules, biotin, or thiol molecules, owing to their accessible 3' and 5' ends, enhancing their versatility in diverse applications [25, 26]. Another significant advantage of aptamers is their exceptional stability in challenging environmental conditions, including high pH, elevated temperatures, and samples containing diverse molecules. This robustness is a key asset in fields such as diagnostics, therapeutics, and biosensing, where the molecular recognition element must maintain its binding affinity and integrity, resulting in a longer shelf life compared to antibodies [27]. As a result, aptamers have emerged as an intriguing alternative to antibodies in biological identification due to their in vitro selections, high specificity, adaptability, stability, and versatility.

Electrochemical detection methods are considered faster and more easily applicable as they do not require complex instrumentation or intricate sample preparation steps. In contrast to radioactive and optical analyses, electrochemical methods are nontoxic and nonradioactive and not sensitive to errors caused by sample turbidity. Biosensors that utilize electrochemical impedance spectroscopy (EIS) for signal transduction have been widely studied in the academic literature, with several concepts. Impedance biosensors are fabricated by immobilizing a biorecognition molecule, such as aptamers, onto a conductive and biocompatible electrode and then detecting the change in the interfacial impedance upon analyte binding by label‐free and direct detection mode [28]. Due to its all‐electrical nature, impedance biosensors are simpler than other methods because they lack optical or acoustic components, offering significant advantages for portable applications [29]. This makes impedance biosensors ideal for clinical monitoring, especially when on‐site and fast detection is needed.

This pioneering study set out with the ambitious goal of swiftly and continuously monitoring ioPTH levels to gauge the success of minimally invasive parathyroid surgery. A notable milestone in this innovative endeavor was the introduction of a novel approach: the selection of a PTH‐specific aptamer using a magnetic bead‐based selection process, marking the first instance of such a method in scientific literature. Following meticulous next‐generation sequencing and rigorous characterization, researchers pinpointed the aptamer sequence demonstrating the highest levels of sensitivity and specificity. This remarkable breakthrough paved the way for its seamless integration into an EIS biosensor system, facilitating real‐time and label‐free detection of PTH. This convergence of cutting‐edge aptamer technology and advanced biosensing techniques heralds a paradigm shift in ioPTH monitoring, offering unprecedented accuracy and efficiency in assessing the outcomes of minimally invasive parathyroid surgery.

## 2 Experimental Section

### 2.1 Materials

In the aptamer selection process, three different fragments of the PTH peptide, PTH (53–84), PTH fragment (1–34), and PTH (1–84), were obtained from Sigma‐Aldrich. PTH (53–84) represents the C‐terminal fragment of PTH, while PTH (1–34) corresponds to the active N‐terminal fragment of PTH. Additionally, to initiate the SELEX procedure, PureProteome 1.0 µm Carboxy FlexiBind Magnetic Beads were procured from Merck, and Hydrophilic Streptavidin Magnetic Beads were obtained from Merck KGaA (Darmstadt, Germany). Bovine serum albumin (BSA), D‐glucose, lactate, human serum albumin (HSA), *N*‐hydroxysuccinimide (NHS), 1‐ethyl‐3‐(3‐dimethylaminopropyl) carbodiimide (EDC), phosphate‐buffered saline (PBS), and potassium ferrocyanide [K_4_Fe(CN)_6_] were all purchased from Sigma‐Aldrich. Carboxylated multi‐walled carbon nanotube powder (MWCNTs‐COOH, > 8% carboxylic acid functionalized, average diameter × L 9.5 nm × 1.5 µm) was purchased by Millipore Sigma, Germany. Human serum (H4522) was purchased from Sigma‐Aldrich Chemie GmbH Export Department (Taufkirchen, Germany). All electrochemical measurements, including cyclic voltammetry (CV) and EIS, were conducted using a PalmSens4 device (PalmSens BV, Netherlands). A standard three‐electrode setup, Carbon Screen Printed Electrode (SPE), was utilized for the electrochemical analysis. In the SPE electrode system, a triple electrode system was employed, comprising a carbon working electrode, a carbon auxiliary (counter) electrode (3 mm in diameter), and a silver/silver chloride reference electrode. Distilled water was employed to prepare the aqueous solutions for all studies conducted in this experiment.

### 2.2 Random Library and Primers

SELEX experiments were conducted following the methods outlined in the literature [30]. For aptamer selection, a DNA library sequence (5'‐AGC AGC ACA GAG GTC AGA TG‐N40‐CCT ATG CGT GCT ACC GTG AA‐3'), along with a forward primer (5'‐AGC AGC ACA GAG GTC AGA TG‐3') and a reverse primer (5'‐TTC ACG GTA GCA CGC ATA GG‐3'), was utilized. Biotin‐ and fluorescence‐labeled primers were commercially synthesized with 5' labeling (RP‐Bio primer [5'‐Biotin‐TTC ACG GTA GCA CGC ATA GG‐3'] and FP_Fl primer [5'‐Fam‐AGC AGC ACA GAG GTC AGA TG‐3']). All of these materials were procured from Ella Biotech GmbH (Germany).

### 2.3 Magnetic Bead Immobilized Peptide Preparation for SELEX

In this study, three distinct fragments of PTH were selected and employed. To initiate the SELEX procedure, the peptides required immobilization on magnetic beads. The EDC/NHS (1‐ethyl‐3‐(3‐dimethylaminopropyl) carbodiimide/*N*‐hydroxysuccinimide) method was utilized to activate the carboxyl groups on the magnetic beads. This involved preparing an EDC solution at a concentration of 40 mM and an NHS solution at a concentration of 100 mM, which were subsequently combined in a 1:1 volume/volume (V/V) ratio.

Magnetic beads (50 µL) were extracted from a stock solution of Carboxy FlexiBind Magnetic Beads (1 mg/mL) using a magnet and were washed three times with PBS. The volume was then adjusted to 50 µL using PBS. Subsequently, 2.5 µL of the prepared EDC/NHS mixture was added to the 50 µL magnetic beads. This mixture was incubated for 2 h at room temperature with constant stirring on a shaker. After incubation, the magnetic beads were separated using a magnet, washed three times with PBS, and the volume was readjusted to 50 µL with PBS.

Following this activation, 50 µL of each PTH fragment (at a concentration of 1 mg/mL) and 1 µL of 1 mg/mL activated magnetic beads were added to the solution. This mixture was incubated by stirring on a shaker for 1 h at room temperature to facilitate the binding of the PTH fragments to the magnetic beads. The binding efficiency of the PTH fragments onto the magnetic beads was subsequently analyzed using a NanoDrop UV‐Vis Spectrophotometer (Thermo Scientific, USA).

### 2.4 In Vitro Selection of Aptamer by SELEX

For the SELEX procedure, a DNA library with a 40‐nucleotide (nt) random region (5'‐AGC AGC ACA GAG GTC AGA TG‐N40‐CCT ATG CGT GCT ACC GTG AA‐3') was utilized. The forward primer sequence was 5'‐AGC AGC ACA GAG GTC AGA TG‐3' and the reverse primer was 5'‐TTC ACG GTA GCA CGC ATA GG‐3'. SELEX proceeded by incubating DNA library pools with target proteins conjugated to magnetic beads. Fifty microliters (µL) of the DNA library was combined with 25 µL of PTHs‐immobilized magnetic beads, followed by adding 200 µL of PBS. The mixture was then incubated by shaking at room temperature for 2 h. Subsequently, magnetic beads were collected using a magnet and used for PCR for binding control (to assess nonspecific binding) and enrichment.

To each peptide‐bound magnetic bead suspension, 25 µL of water was added. For a 50 µL PCR sample, 5 µL of the peptide‐bound magnetic beads were then added from the previously prepared mixture (10X Taq Buffer, 25 mM MgCl_2,_10 mM dNTP, 10 µM Forward Primer, 10 µM Reverse Primer, 5 U/µL Taq DNA Polymerase). The volume was adjusted to 50 µL by adding pure water, and PCR was performed. After completion of the process, the samples were checked for molecular weight by conducting an optimized low‐range ultra‐agarose gel electrophoresis experiment.

The PCR products obtained after each SELEX cycle were initially separated into single‐stranded DNA (ssDNA) by PCR amplification using 5ʹ‐biotin‐labeled reverse primers. The double‐stranded PCR products were mixed with streptavidin‐coated magnetic beads (Sigma Aldrich, USA) in PBS and incubated for 1 h. Subsequently, the non‐biotinylated ssDNA strands were separated from the beads by heating at 95°C for 2 min. The resulting ssDNA library was then collected for use in the subsequent cycle, serving as the next round library. This procedure was reiterated until the 12th cycle to acquire sequences that specifically bind to the target peptides. Furthermore, a negative selection was carried out by adding BSA at a concentration of 1 mg/mL to each incubation mixture starting from Round 5. This step aimed to minimize the potential binding of nonspecific oligonucleotides to proteins.

### 2.5 Next‐Generation Sequencing and Structural Analysis of Aptamer

After completing 12 cycles of SELEX, specifically binding sequences were prepared by re‐amplification with NGS forward and NGS reverse primers. The sequences obtained were categorized into groups based on shared motif regions using the MEME SUITE program. They are evaluated for specific binding to the target peptide, and the aptamer candidate for each target peptide was determined. The aptamers’ secondary structures of the ssDNA molecules were also predicted using the Mfold web server [31, 32].

### 2.6 Aptamer‐Protein Binding Assay for Characterization

**FIGURE 1:**
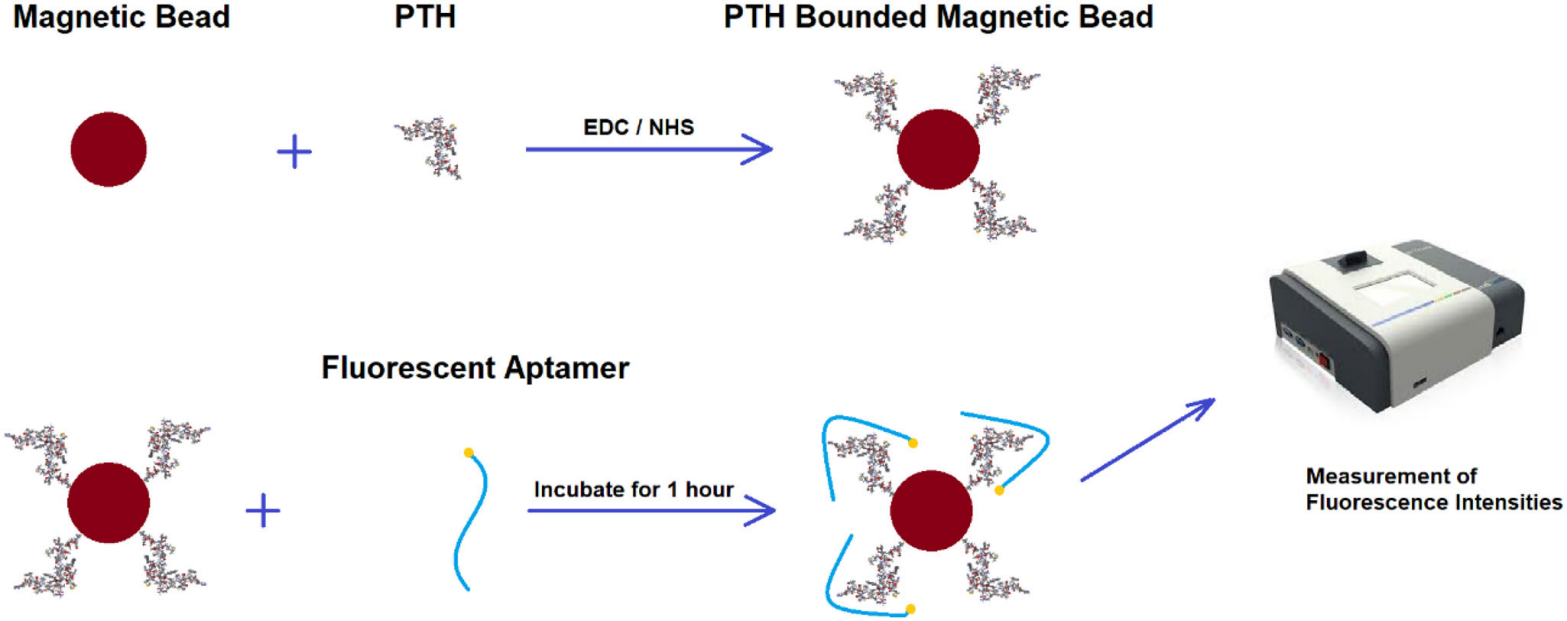
Schematic of the characterization test for evaluating the PTH binding sensitivities of candidate aptamers obtained from SELEX cycles using the fluorescence‐based method.

Fluorescently labeled forms of the aptamer, selected from sequences containing the determined motif, were used for aptamer characterization. The binding rates of these motif sequences to different regions of the PTH hormone, bound to magnetic beads prepared under SELEX conditions, were determined at various aptamer concentrations ranging from 100 to 2500 nM. After mixing aptamers with magnetic beads and incubating for half an hour, washing was performed, and fluorescence intensities were measured. The aptamer exhibiting the lowest *K*_d_ value, indicating the most sensitive binding, was selected for each PTH hormone. Subsequently, the aptamer with the lowest *K*_d_ value among the three peptides was utilized for the impedimetric biosensor study (Figure 1).

### 2.7 Impedimetric Biosensor Study

**FIGURE 2:**
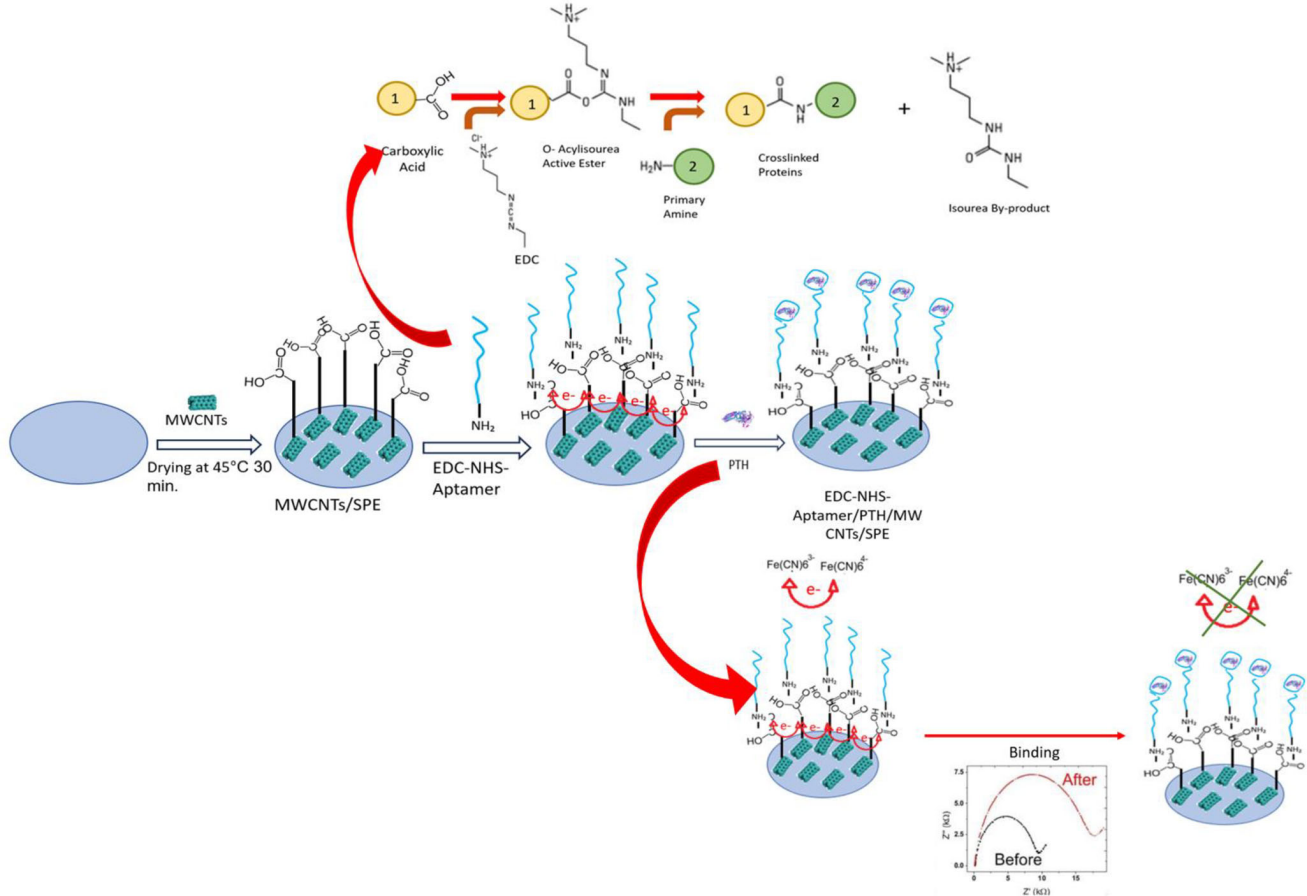
Schematic view of aptamer‐based electrochemical biosensor system. CV and EIS measurements were performed on a bare electrode to obtain the aptamer's Nyquist plots and calibration curve. MWCNT was drop‐cast onto the electrodes and dried at 45°C. After EDC/NHS activation, aptamer immobilization was carried out. The electrodes were tested with the target analyte at concentrations ranging from 2 to 600 pg/mL.

The detailed working principles of the impedimetric biosensor system for PTH detection are presented in Figure 2. The aptamer‐based impedimetric biosensor was fabricated through a three‐step process. First, the electrode surface was modified with MWCNTs. Second, the MWCNT‐modified electrode was functionalized for aptamer immobilization. Finally, the biosensor was used for PTH detection via EIS. The binding of PTH to the immobilized aptamer on the electrode surface is expected to induce a change in the measured impedance, thereby allowing for the determination of PTH presence and concentration in the sample.

In the modification of the screen‐printed electrode (SPE) surface with carbon nanotubes, a 10 µg/mL concentration of carboxylic acid‐modified MWCNTs was first dissolved in a NaCl solution using an ultrasonic stirrer. Then, 10 µL of the dissolved carbon nanotubes were deposited on the electrode surfaces. The MWCNT film was allowed to dry at room temperature for 1 day before proceeding to the next steps. Subsequently, an amino‐modified PTH aptamer was attached to the MWCNT‐modified electrode surface using EDC/NHS linkers. The EDC crosslinker activates carboxyl groups, forming an unstable intermediate called O‐acylisourea, which reacts with primary amines to form stable amide bonds. NHS or Sulfo‐NHS is added to stabilize this reaction. In this protocol, carboxylic acid‐modified MWCNTs and amino‐modified aptamer were covalently linked using EDC/NHS chemistry (Figure 2). EDC activates carboxyl groups, allowing them to form covalent bonds with primary amines in a manner akin to C‐terminal bioconjugation [33, 34].

Following the immobilization of aptamers onto the SPE surface, an experimental protocol was systematically executed to assess the biosensor's performance across a range of PTH concentrations. Seven distinct PTH concentrations (2, 20, 50, 100, 200, 400, and 600 pg/mL) were precisely applied to the sensor surface to allow for specific binding interactions with the immobilized aptamers. After a 30‐min incubation period at ambient temperature to ensure optimal binding kinetics, the SPE surface was washed with PBS and subjected to EIS measurement. The binding was monitored by measuring the change in charge transfer resistance (Rct) before and after PTH binding to the specific aptamer. The differences in Rct values for each PTH concentration were calculated and used for further analysis.

During the biosensor preparation and PTH detection steps, the EIS was recorded over a frequency range from 10 kHz to 0.1 Hz using an alternative voltage with an amplitude of 10 mV, superimposed on a DC potential of 0.1 V (vs. an Ag/AgCl reference electrode). The impedance data were plotted in the form of a complex plane diagram (Nyquist plot), and fitted to a modified Randles equivalent circuit. In addition to the EIS analysis, the CV experiments were conducted at a scan rate of 100 mV/s. All the EIS and CV measurements were recorded in a 0.1 M KCl solution containing 5 mM [Fe(CN)_6_]^−3/−4^ redox pair (1:1 molar ratio).

For constructing the linear calibration curve for impedimetric PTH detection, each experiment was performed at least three times, and standard deviation (SD) values for each concentration were calculated. To evaluate the system's repeatability and reusability, the aptamer‐immobilized SPE surface was tested with 200 pg/mL PTH concentration five times, performing an elution step after each test.

For selectivity studies, 200 pg/mL of PTH was mixed with the same concentration of other molecules commonly present in the blood, such as human serum albumin (HSA), lactate, glucose, and urea, and tested with the developed impedimetric aptasensor system. Additionally, a 200 pg/mL concentration of PTH was mixed with real human serum in a 1:5 ratio and tested to observe the matrix influence of actual samples on the biosensor performance after initially optimizing the working parameters.

This rigorous experimental approach enabled the characterization of the biosensor's sensitivity and selectivity across a wide dynamic range of PTH concentrations and provided valuable insights into the kinetics of the aptamer‐PTH interaction under physiologically relevant conditions.

## 3 Results and Discussion

### 3.1 SELEX and Aptamer Characterization for PTH

In the initial part of the SELEX process, the binding of peptides to magnetic beads was demonstrated by determining the peptide concentration based on the maximum absorbance at 280 nm using the NanoDrop UV‐Vis spectrophotometer. As depicted in Figure S1, the analysis of the supernatant obtained after binding the peptides to the magnetic beads shows a decrease in peptide concentration, as indicated by the A280 nm absorbance. This decrease signifies that some peptides have bound to the magnetic beads, with additional peptides being removed during washing. Specifically, approximately 0.118 mg/mL of the PTH (53–84) peptide, 0.268 mg/mL of the PTH (1–84) peptide, and 0.353 mg/mL of the PT (1–34) peptide were found to be fixed to the magnetic beads. The differences in fixation rates can be attributed to the hydrophobicity values of the peptide fragments and their interactions with the magnetic surface. Several studies suggest that the critical factors influencing the bio‐nano bonding interface include the structural shapes, sizes, charges, and the effects of the bonding mixture [35, 36, 37, 38].

**FIGURE 3:**
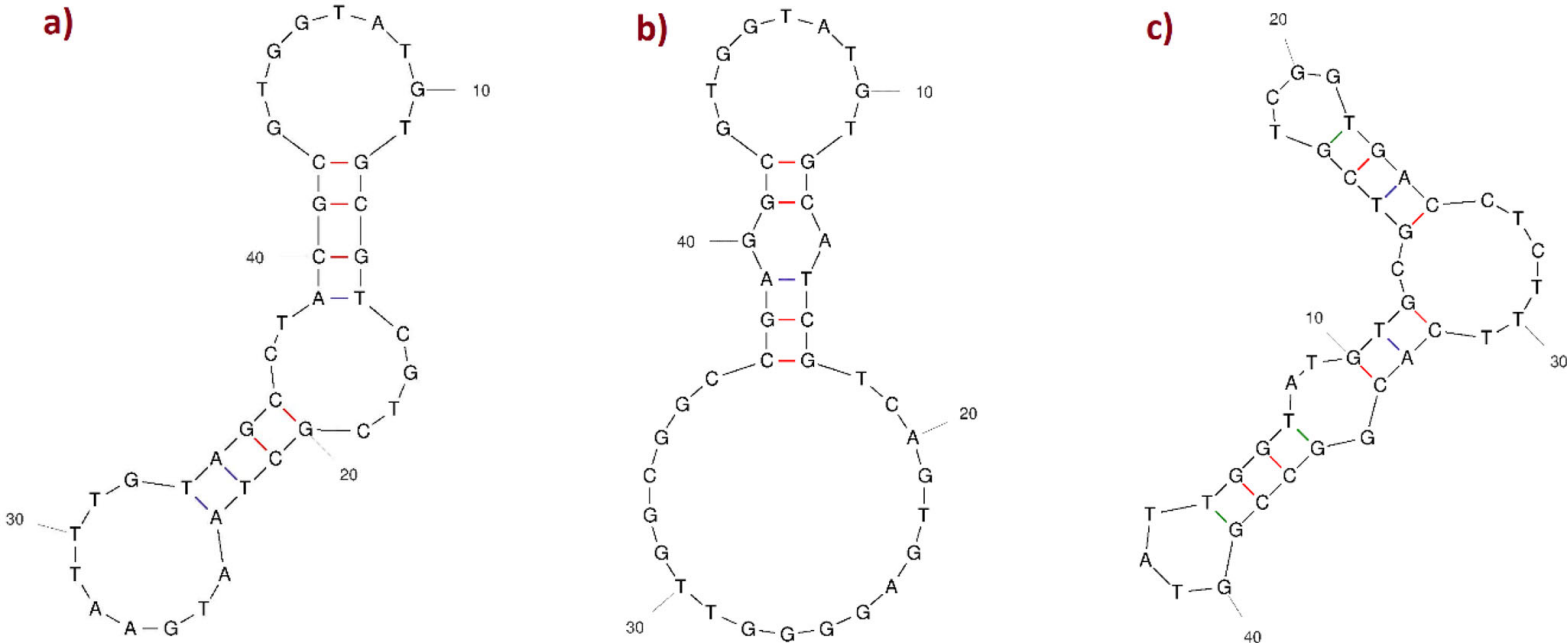
The predicted secondary structures of the three aptamers from the Mfold web server: (A) aptamer—PTH (53–84); (B) aptamer—PTH (1–34); and (C) aptamer—PTH (1–84).

The DNA library was combined with 25 µL of PTH‐immobilized magnetic beads, followed by the addition of 200 µL of PBS. The mixture was then incubated with shaking at room temperature for 2 h. Subsequently, the magnetic beads were collected using a magnet and subjected to PCR for binding control, to assess nonspecific binding, and for enrichment. The PCR amplifications and molecular weights were verified through agarose gel electrophoresis, and the results were captured using the ChemiDoc Imaging System (Bio‐Rad). Figure S2 displays the gel electrophoresis outcomes of the samples following the 12th SELEX cycle. After a successful 12 SELEX cycle, after the final cycle, it was observed that PCR products, which exhibited stronger binding affinity to negative selection molecules compared to libraries enriched with peptides, were subjected to re‐amplification using NGS forward and NGS reverse primers. Subsequently, next‐generation sequencing was conducted and the obtained sequences were divided into groups according to common motif regions with the MEME SUITE program. A total of 14,227 sequences were identified for PT 53–84 through this sequencing process. Motif analysis revealed the presence of a six‐base motif (TATGTG) in 4327 sequences, constituting 30.4% of the total. Similarly, for PT 1–34, a seven‐base motif (TGAGGGG) was identified in 12,627 sequences, with 5327 sequences (42.2%) containing this motif. Regarding PT 1–84, an eight‐base motif sequence (TGCGTCGT) was observed in 2866 out of 9621 total sequences, representing 29.7% of the sequences. Consequently, the group with the highest number of sequences sharing a common motif was chosen as the aptamer candidate for each target peptide [39, 40]. Motif analysis revealed that apart from the dominant motifs mentioned above, all other identified motifs had an abundance of less than 15%. Detailed information on these low‐abundance motifs is provided in the Supporting Information (Table S1). Among these candidates, a fluorescent binding assay was performed to identify the aptamer with the lowest *K*_d_ value, which was then selected for use in the experiments as the representative for that peptide (Table 1). The predicted secondary structures of the three aptamers are also depicted in Figure 3. The aptamers’ secondary structures of the ssDNA molecules were predicted using the Mfold web server [31, 32].

**TABLE 1:** Sequences identified as aptamer candidates for each peptide based on motif identification.

**Aptamer‐PTH (53–84)**	GCGTGGTATGTGCGTCGTCGCTAATGAATTTGTAGCCTAC
**Aptamer‐PTH (1–34)**	GCGTGGTATGTGCATCGTCAGTGAGGGGTTGGCGGCCGAG
**Aptamer‐PTH (1–84)**	TATTGGTATGTGCGTCGTCGGTGACCTCTTTCACGGCCGG

**FIGURE 4:**
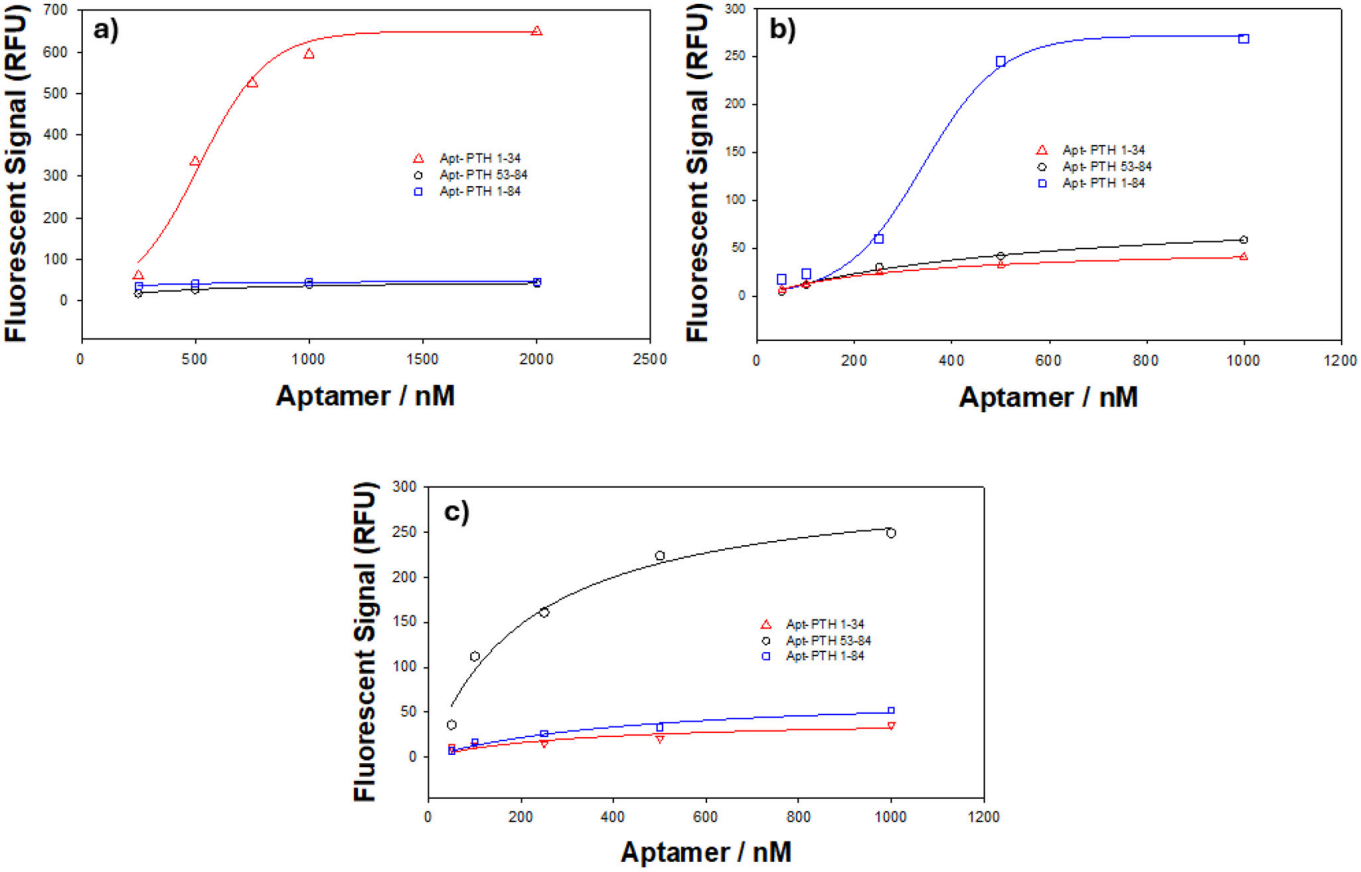
Results of characterization (affinity) analysis of aptamer sequences: (A) PTH (1–34), (B) PTH (1–84), (C) PTH (53–84).

Fluorescently labeled forms of the aptamer, selected from sequences containing the determined motif, were used for aptamer characterization via fluorescent binding assay. The binding rates of these motif sequences to different fragments of the PTH hormone, bound to magnetic beads prepared under SELEX conditions, were determined at various aptamer concentrations ranging from 100 to 2500 nM. To ensure specificity, aptamers of different PTH fragments were used as negative controls in the binding assays. The fluorescence intensity measurements at different aptamer concentrations allowed for the determination of binding affinity constants (*K*_d_) and provided insights into the aptamer's specificity and binding efficiency. Figure 4 presents the results of the molecular binding analysis of the aptamer candidate sequences with different PTH fragments. The binding experiments were conducted using a fixed concentration of the magnetic bead‐peptide complex while varying the concentrations of fluorescently labeled aptamers. The resulting binding curves were analyzed using the Langmuir single‐analyte binding model (SigmaPlot 11), revealing distinct binding affinities among the three aptamers. The calculated binding affinity constants (*K*_d_) were 515.8 ± 41.0 nM for the PTH (1–34) aptamer, 339.7 ± 23.3 nM for the PTH (1–84) aptamer, and 220.5 ± 57.5 nM for the PTH (53–84) aptamer. These values indicate that the PTH (53–84) aptamer exhibits the strongest binding affinity, followed by PTH (1–84) and PTH (1–34), respectively.

In Figure 4A, the PTH (1–34) aptamer (red triangles) exhibits a moderate binding interaction, as indicated by its fluorescence signal, which increases with aptamer concentration and reaches a saturation plateau at higher concentrations. However, its higher *K*_d_ value suggests a relatively weaker binding affinity. In Figure 4B, the PTH (1–84) aptamer (blue squares) displays a stronger binding interaction, as evidenced by a more pronounced fluorescence increase, confirming a higher affinity than the PTH (1–34) aptamer. Finally, Figure 4C illustrates that the PTH (53–84) aptamer (black circles) generates the highest fluorescence signal among the three, reaching saturation at lower aptamer concentrations, which is consistent with its lowest *K*_d_ value and superior binding affinity. These results confirm that the PTH (53–84) aptamer exhibits the strongest and most specific interaction with its target fragment, the C‐terminal region of PTH. The use of the PTH (1–34) and PTH (1–84) fragments as negative controls further validates the specificity of the PTH (53–84) aptamer, as their fluorescence intensities remained significantly lower in binding experiments.

Considering both the fluorescence binding curves and *K*_d_ values, the PTH (53–84) aptamer is the most effective candidate for PTH detection. Its combination of the lowest *K*_d_ value (220.5 ± 57.5 nM) and the highest fluorescence intensity suggests its strong and specific binding characteristics, making it particularly suitable for biosensing applications requiring high sensitivity and specificity. This aptamer's superior performance highlights its potential utility in biosensor platforms designed for intraoperative PTH analysis, where precise and real‐time monitoring of PTH levels is critical for surgical decision‐making. Consequently, the PTH (53–84) aptamer sequence was incorporated into the impedimetric biosensor study to facilitate the accurate detection of PTH in pre‐ and postoperative clinical assessments.

### 3.2 Imedimetric Biosesensor for PTH Detection

This section presents the comprehensive results of the impedimetric biosensor study conducted to detect PTH, highlighting the sensitivity, specificity, and reliability of the developed biosensing system.

**FIGURE 5:**
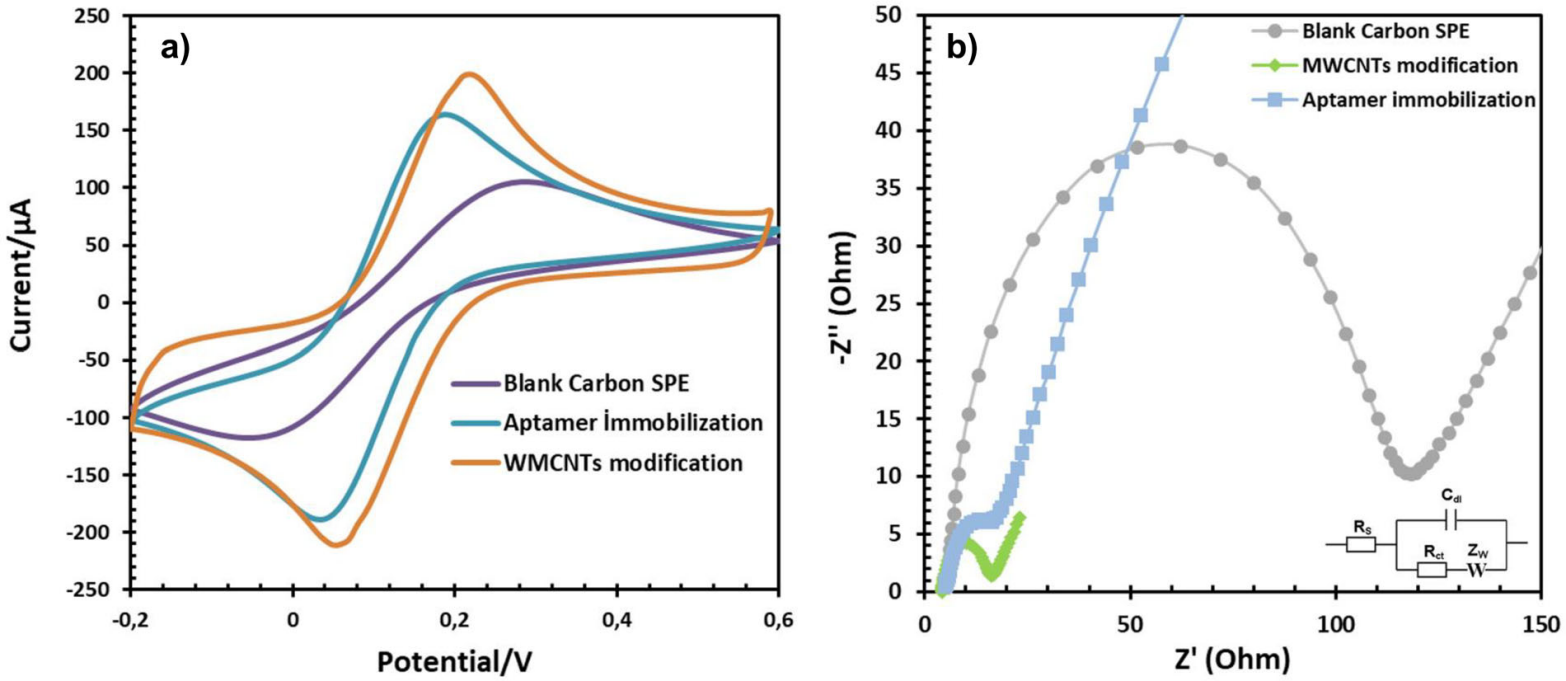
(A) Cyclic voltammograms of the electrode at various stages of the aptasensor construction were recorded at a scan rate of 50 mV/s in a solution of PBS (0.1 M, pH 7.4) containing 5 mM K_4_[Fe(CN)_6_]. (B) Nyquist plots of the following electrodes were obtained in 0.1 M PBS (pH 7.4) containing 5 mM K4[Fe(CN)6] in the frequency range of 100 kHz to 10 mHz.

In the initial phase of the biosensor preparation process, both CV and EIS measurements were employed to scrutinize the modification of the SPE surface with MWCNTs and the subsequent covalent immobilization of the aptamer onto it. Notably, the introduction of MWCNTs to the SPE yields a discernible augmentation in the peak current density of the redox probe, as evidenced by the CV measurement (Figure 5A, orange curve). This enhancement can be attributed to the exceptional electrical properties of MWCNT material, which notably expedite the rate of electron transmission owing to its high conductivity and extensive specific surface area.

Subsequent to the covalent immobilization of the aptamer onto the MWCNT‐coated SPE, a conspicuous decrease in the redox peak current density with CV measurement and an accompanying elevation in separation potential were observed (Figure 5A, blue curve), in comparison to the MWCNTs/SPE configuration (Figure 5A, orange curve). This phenomenon is logically explained by the fact that the presence of the aptamer, *N*‐hydroxysuccinimide (NHS), and the non‐conductive 1‐ethyl‐3‐(3‐dimethylaminopropyl) carbodiimide (EDC) collectively form an insulating barrier, impeding the interfacial charge transfer process.

In the EIS measurements, the Nyquist plot of the bare SPE (Figure 5B, grey curve) reveals a small semicircle, indicative of low charge transfer resistance (Rct) values, implying a clean SPE surface with efficient charge transfer. Upon surface modification with MWCNTs (Figure 5B, green curve), the Rct value decreases compared to that of the bare SPE, reflecting enhanced conductivity at the electrode–electrolyte interface. However, upon immobilization of the aptamer onto the MWCNTs/SPE surface, the Rct increases, signifying the formation of a non‐conductive layer that impedes charge transfer between the aptasensor's surface and the redox probe (Figure 5B, blue curve). These EIS results are consistent with the CV findings, providing complementary evidence of the electrode surface modifications and their influence on charge transfer characteristics.

After the preparation of the MWCNTs and aptamer‐modified SPE surfaces, the electrodes were incubated for 30 min with seven distinct PTH solutions at varying concentrations: 20, 50, 100, 200, 400, and 600 pg/mL. The EIS responses were recorded in a solution of 0.1 M KCl containing 5 mM potassium ferricyanide (K_4_[Fe(CN)_6_]) to comprehensively analyze the performance of the aptasensors (see Figure 5A). A notable finding is the direct relationship between the concentration of PTH and the charge transfer resistance (Rct) values. The increase in Rct values is attributed to the modification of the aptamer's spatial arrangement upon interaction with PTH. At higher concentrations of PTH, the aptasensor surface experiences a gradual obstruction of electron transmission due to the increasing formation of PTH/aptamer complexes. This inhibitory phenomenon primarily arises from the blockage created by the bound PTH molecules, which impedes efficient electron transfer to the aptasensor's surface.

**FIGURE 6:**
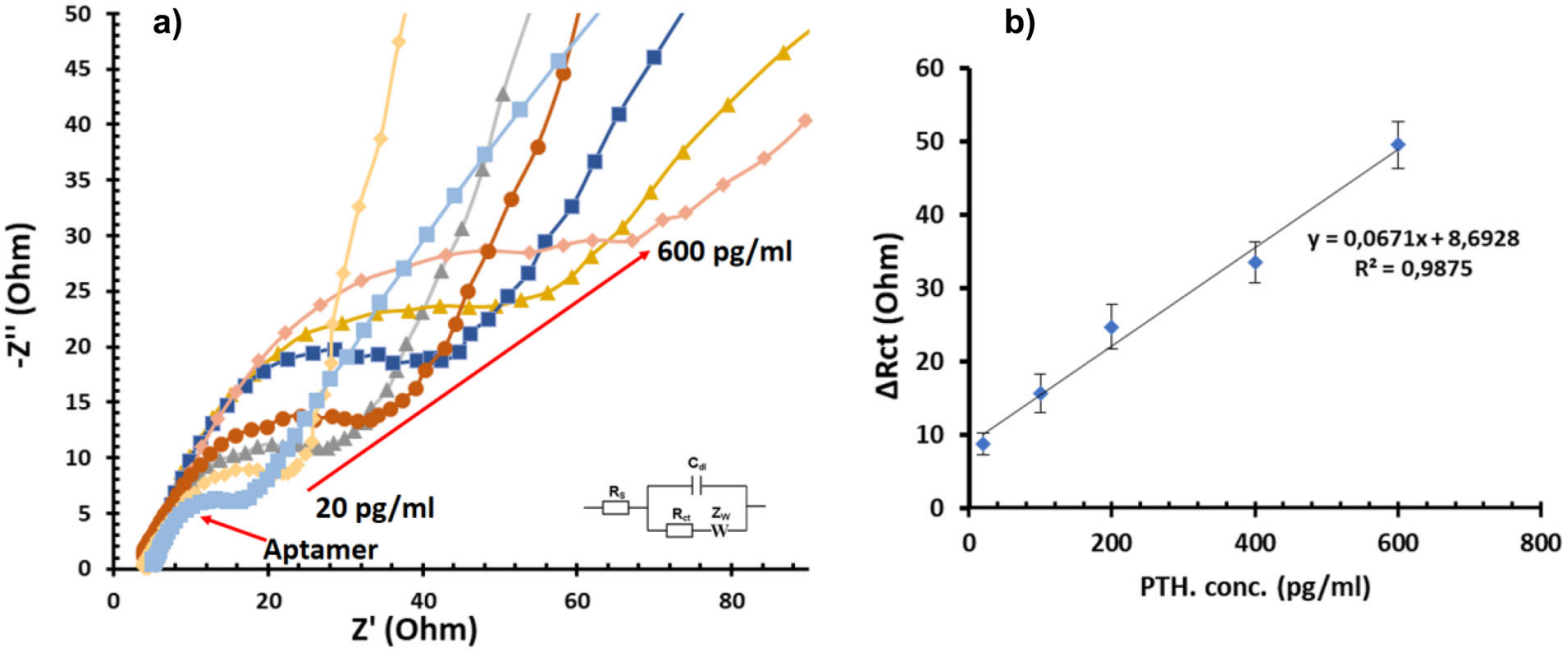
(A) Nyquist plots were obtained in 0.1 M PBS containing 5 mM K_4_[Fe(CN)_6_] after incubating the aptasensor with different concentrations of PTH solutions ranging from 2 to 600 pg/mL. (B) Calibration curve using Rct versus Log PTH concentration.

Next, a calibration curve was derived from the EIS data presented in Figure 6. Figure 6B illustrates the variations in charge transfer resistance (Rct) with different concentrations of PTH. The data reveal a specific range where Rct increases linearly with the concentration of PTH, spanning from 20 to 600 pg/mL. The linear regression equation for this relationship is Rct = 0.0671 [PTH] + 8.6928, with an impressive correlation coefficient (*R* ^2^) of 0.9875. This high *R* ^2^ value indicates a strong linear correlation between the PTH concentration and the charge transfer resistance, demonstrating the aptasensor's high sensitivity and reliability for quantifying PTH levels. Furthermore, the experimental detection limit was determined to be 20 pg/mL, underscoring the aptasensor's capability for detecting even low concentrations of PTH. The linearity, precision, and low detection limit of this calibration curve validate the aptasensor's potential for accurate PTH detection in clinical and biomedical applications.

Selectivity is an essential characteristic that allows accurate analyte detection without giving in to the effect of other components or interferents. In the field of biosensor design, achieving a high degree of selectivity for PTH measurement constitutes an important goal. This goal can be accomplished by enhancing the analyte's interaction with a particular chemical element while simultaneously limiting the possible negative effects of potential substitute interferents [41, 42].

**FIGURE 7:**
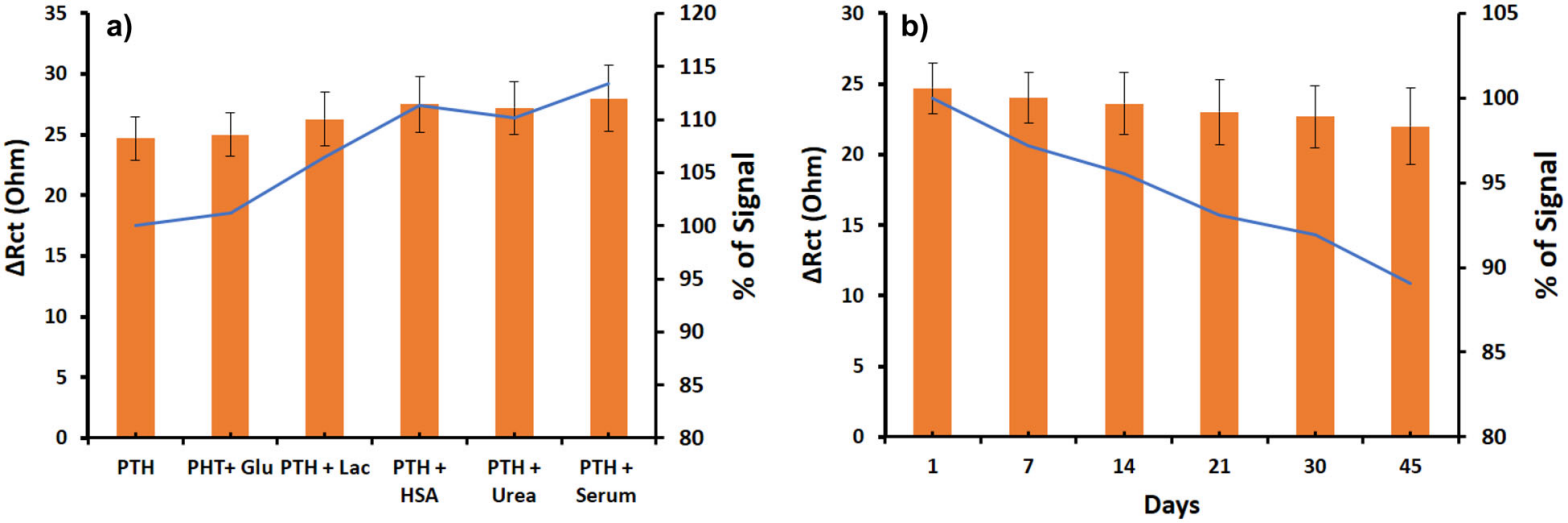
(A) EIS results of the aptamer‐based biosensor's selectivity with glucose, urea, HSA, lactate, and also the addition of human serum. (B) Stability of the aptamer‐based biosensor systems from Day 1 to Day 45 under the same conditions.

To assess the selectivity of the biosensor system designed for PTH detection, glucose, urea, human serum albumin (HSA), and lactate were employed as nonspecific analytes. These analytes were mixed with a 200 pg/mL PTH solution and subsequently introduced to the electrode surface after aptamer modification. The biosensor's responses to these mixtures were meticulously monitored through EIS measurements. As depicted in Figure 7A, the EIS signals demonstrate the remarkable selectivity of the biosensor system. The signal variations observed in response to the introduction of nonspecific analytes ranged from 8% to 12%, indicating minimal interference and highlighting the system's specificity for PTH detection. This high selectivity underscores the biosensor's potential for accurate PTH measurement in complex biological samples.

The developed aptamer‐based biosensor system was further evaluated using real serum sample analyses. PTH‐spiked real serum samples were introduced onto the aptamer‐modified electrodes to assess the system's performance. Specifically, PTH concentrations of 200 pg/mL were mixed into one‐fifth diluted real human serum samples and sequentially introduced to the biosensor. During this phase of the performance test, EIS measurements were conducted for the mixture, similar to the procedure used with other interference molecules. The biosensor system demonstrated exceptional accuracy, achieving a recovery rate of up to 113%. Additionally, the SD percentage for accurately detecting PTH was 9.6%, indicating high precision. These results are depicted in Figure 7A, highlighting the biosensor's capability for precise PTH detection in complex biological matrices.

The biosensor systems, prepared using aptamer‐modified SPE surfaces, underwent a stability test by storing them at 4°C for 45 days. Regular PTH tests were conducted during this period with a concentration of 200 pg/mL, and the EIS results were obtained. Subsequently, the acquired results were evaluated in comparison with the initial data to assess the stability of the systems. As illustrated in Figure 7B, the stability of the results obtained with the developed aptamer‐based biosensor system was determined to be 89% after 45 days. These findings provide evidence that the system maintains a high level of stability even under prolonged storage conditions, ensuring reliable and consistent performance over an extended period.

The ability of a biosensor to produce consistent results under identical conditions is referred to as repeatability. During the stability assessment of the developed biosensor system, each experiment was performed at least five times, and the system's repeatability was evaluated by calculating SD of the results. The percentage of SD observed ranged from 7.2% to 9.6% for the aptamer‐modified SPE surfaces. This range indicates a high degree of precision and reliability in the biosensor's performance, affirming its suitability for consistent PTH detection in practical applications.

## 4 Conclusion

In conclusion, this study presents the development of an aptamer‐based electrochemical biosensor tailored for real‐time monitoring of PTH levels, with a particular focus on intraoperative assessment during parathyroid surgery. The research encompassed the selection and characterization of aptamers targeting distinct segments of the PTH peptide, enabling comprehensive detection across various PTH isoforms. Through a meticulously designed experimental framework, which included aptamer selection via SELEX, aptamer–peptide interaction analysis, and biosensor fabrication, the study successfully demonstrated the feasibility and efficacy of the proposed biosensing platform. The SELEX process yielded aptamers with notable binding affinities to different regions of PTH, with the PTH (53–84) aptamer exhibiting particularly sensitive binding to the hormone's C terminus, thus offering the potential for precise PTH analysis.

Electrochemical characterization of the biosensor unveiled substantial alterations in impedance signals upon exposure to varying concentrations of PTH, providing valuable insights into the sensor's sensitivity and selectivity. The observed increase in charge transfer resistance (Rct) values with escalating PTH concentrations underscored the biosensor's capability to detect PTH‐induced structural changes, validating its potential for accurate PTH measurement. Moreover, the establishment of a calibration curve based on EIS data further affirmed the biosensor's performance, showcasing a linear relationship between Rct values and PTH concentrations. The regression equation derived from this curve provided a quantitative means of determining PTH levels, with high correlation coefficient (*R* ^2^) values attesting to the reliability of the biosensor's measurements.

The biosensor exhibited remarkable selectivity in the presence of common interferents found in serum samples, ensuring precise detection of PTH. Furthermore, stability assessments over a 45‐day storage period demonstrated the robustness and long‐term reliability of the biosensor system, affirming its suitability for practical applications.

In summary, the developed aptamer‐based biosensor represents a promising tool for sensitive and selective detection of PTH. It has potential applications in biomedical research and clinical diagnostics, particularly intraoperative PTH analysis during parathyroidectomy procedures. Continued research and optimization endeavors promise to enhance the biosensor's performance and expand its utility across diverse healthcare settings.

## Author Contributions

**Reza Didarian**: methodology, writing–original draft preparation. **Saharnaz Bargh**: resources, software. **Almina Gülerman**: methodology. **Veli Cengiz Özalp**: reviewing and editing, conceptualization, resources. **Özcan Erel**: reviewing and editing. **Nimet Yildirim‐Tirgil**: methodology, writing–original draft preparation, reviewing and editing, project administration, supervision.

## Disclosure

During the preparation of this work, the authors used the ChatGPT AI program in order to check the grammar and advance the English writing of the manuscript. After using this tool/service, the authors reviewed and edited the content as needed and take full responsibility for the content of the publication.

## Supporting information



Figure S1. Analysis of peptides with NanoDrop UV‐Vis Spectrophotometer: (A) PTH (1–34), (B) PTH (1–84), and (C) PTH (53–84). (a) Analysis of the supernatant after peptide binding to magnetic beads, and (b) analysis of the peptide in its pure form.
Figure S2. The gel electrophoresis results of the samples: (1) PCR result for PT (53–84), (2) PCR result for PT (1–34), and (3) PCR result for PT (1–84).
Table S1. The sequences were obtained with the SELEX procedure for fragments of PTH.
