# Supplementary material for: Selection of DNA Aptamers Against Parathyroid Hormone for Electrochemical Impedimetric Biosensor System Development
Source: Biotechnol Appl Biochem. 2025 Mar 19;72(5):1351–62. doi: 10.1002/bab.2745 (PMC12529457; doi:10.1002/bab.2745)
Supplement: Supplementary file 1 — Figure S1. Analysis of peptides with NanoDrop UV‐Vis Spectrophotometer: (A) PTH (1–34), (B) PTH (1–84), and (C) PTH (53–84). (a) Analysis of the supernatant after peptide binding to magnetic beads, and (b) analysis of the peptide in its pure form. Figure S2. The gel electrophoresis results of the samples: (1) PCR result for PT (53–84), (2) PCR result for PT (1–34), and (3) PCR result for PT (1–84). Table S1. The sequences were obtained with the SELEX procedure for fragments of PTH. [file BAB-72-1351-s001.docx]

**
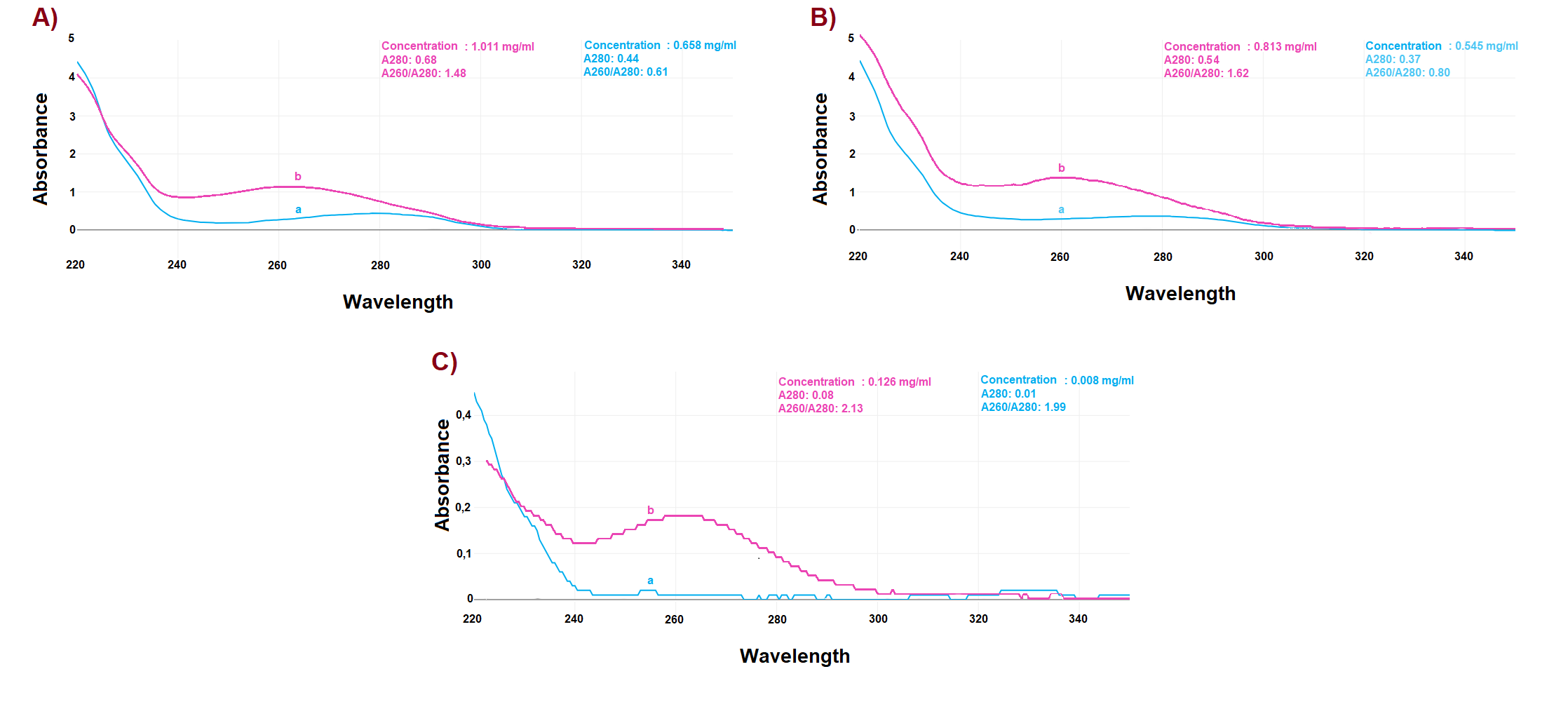
**

**Figure S1. Analysis of Peptides with NanoDrop UV-Vis Spectrophotometer; A) PTH (1-34), B) PTH (1-84), and C) PTH (53-84). a) Analysis of the supernatant after peptide binding to magnetic beads and b) Analysis of the peptide in its pure form."**


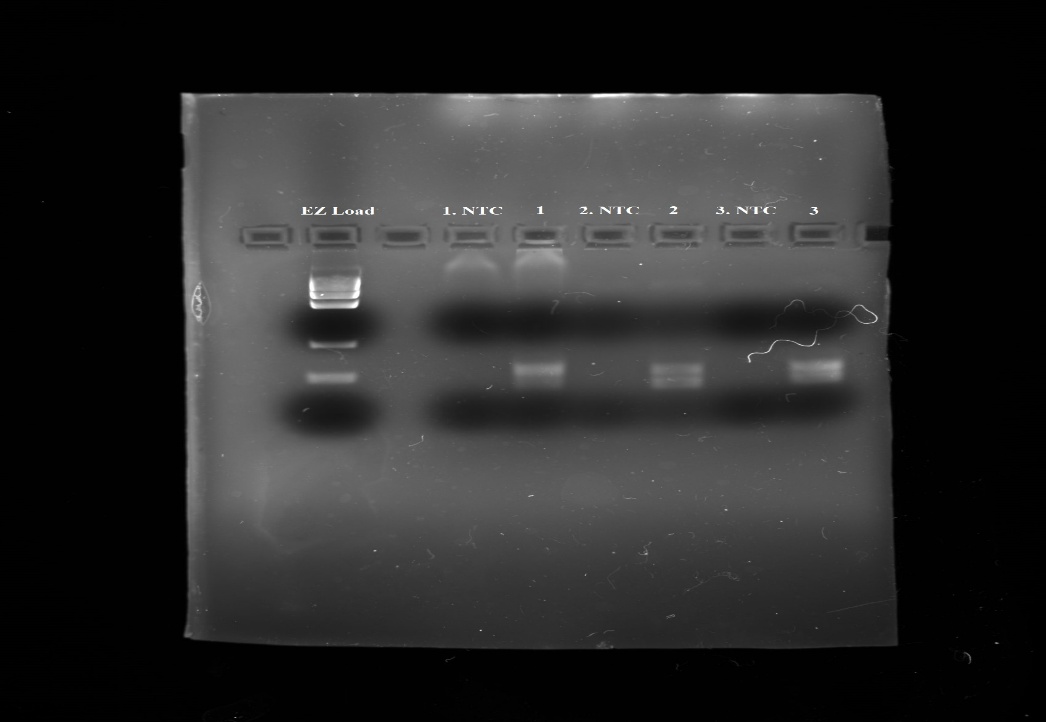


***Figure S2. The gel electrophoresis results of the samples: 1) PCR result for PT (53-84), 2) PCR result for PT (1-34), and 3) PCR result for PT (1-84).***

***Table S1. The sequences obtained with SELEX procedure for fragments of PTH.***

| **Motif** | **Representative Sequence and the motif** | **Abundance (%)** |
| --- | --- | --- |
| PTH (53-84) Motif 1 | GCGTGGTATGTGCGTCGTCGCTAATGAATTTGTAGCCTAC | 30.4 |
| PTH (53-84) Motif 2 | TCCTTATTGCCTAGAATTACTTACTACGGTTAACCATACC | 14.8 |
| PTH (53-84) Motif 3 | CTATTAGTACAGTAGTGCCTTGAATGACATTCTCTTTATT | 6.1 |
| PTH (53-84) Motif 4 | AAGTTTTCTCTACAGCTAAACGATCAAGTGCACTTCCACA | 3.2 |
| PTH (53-84) Motif 5 | TTCGATTATCGCGGCCACTCTCGCATTAATCGGTAGAAGT | 3.1 |
| PTH (1-34) Motif 1 | GCGTGGTATGTGCATCGTCAGTGAGGGGTTGGCGGCCGAG | 42.2 |
| PTH (1-34) Motif 2 | GGTTGTGTTGCGATAGCCCAGTATAATATTCTAAGGTGTT | 14.8 |
| PTH (1-34) Motif 3 | GGGACTAAAGAGGCGATGATAATCGTGAGTGCCGCGTTAT | 10.2 |
| PTH (1-34) Motif 4 | GGTGGTGTCGGAACAGAGCGGTCTTACGGCCAGTCGTATG | 4.3 |
| PTH (1-34) Motif 5 | CCTTCTCGAGCTCCGTCCGGTTAAGCGTGACAGCCCCAGC | 4.1 |
| PTH (1-84) Motif 1 | TATTGGTATGTGCGTCGTCGGTGACCTCTTTCACGGCCGG | 29.7 |
| PTH (1-84) Motif 2 | GAGCGCGGTGGAGACTCGTCCACCCGGCAGCTCTGTAACA | 13.9 |
| PTH (1-84) Motif 3 | ACCCTGATGAATATTCAACAGAATTGCTATAGGCCTTGAA | 11.2 |
| PTH (1-84) Motif 4 | TCAAAAGGTTATACTCTTGTAGTTAACATGTAGCCCGGCC | 9.3 |
| PTH (1-84) Motif 5 | CACTACACGGATGATACGAAATTATGTATAGACCGGGTCA | 2.4 |
